# Clinical outcome of transperitoneal ureterocutaneostomy using the transverse mesocolon

**DOI:** 10.3892/mco.2013.117

**Published:** 2013-05-09

**Authors:** NOBUKI FURUBAYASHI, TAKAHITO NEGISHI, EIJI KASHIWAGI, ATSUSHI FUKUDA, MOTONOBU NAKAMURA

**Affiliations:** Department of Urology, National Kyushu Cancer Center, Fukuoka 811-1395, Japan

**Keywords:** transperitoneal ureterocutaneostomy, transverse mesocolon, urinary diversion, clinical outcome

## Abstract

The aim of the present study was to demonstrate that the treatment outcome of transperitoneal ureterocutaneostomy using the transverse mesocolon is not inferior to that of conventional retroperitoneal ureterocutaneostomy. The enrolled subjects were 26 patients who underwent ureterocutaneostomy among a total of 160 cases of urinary diversion performed at our institution between March, 2004 and November, 2011. A total of 11 cases and 18 ureters were treated by transperitoneal ureterocutaneostomy via the transverse mesocolon, with 7 bilateral ureterocutaneostomy cases. All the cases of retroperitoneal ureterocutaneostomy were unilateral, totaling 15 patients and 15 ureters. Postoperative adverse events (ileus, acute pyelonephritis), catheter-free status and renal function [blood urea nitrogen (BUN) and serum creatinine (Cr) values] were retrospectively evaluated between the groups treated by the different surgical procedures. Ileus was only observed in 1 case and improvement was achieved through conservative therapy alone (P=0.827). Acute pyelonephritis developed in 3 (27.3%) transperitoneal and 8 (53.3%) retroperitoneal cases, with all the cases recovering following treatment with antibiotics alone (P=0.199). A catheter-free status was achieved in 3 cases (27.3%) and 4 ureters (22.2%) in the transperitoneal group and in 2 cases and 2 ureters (13.3%) in the retroperitoneal group (P=0.393). There was no significant change in renal function (BUN and serum Cr values) in the transperitoneal ureterocutaneostomy cases at the postoperative, early postoperative (1 month following surgery) or the final follow-up examination (P=0.739 and 0.078). In conclusion, transperitoneal ureterocutaneostomy allows for the construction of a cutaneous stoma using a shorter ureter, with a treatment outcome that is comparable to that of retroperitoneal ureterocutaneostomy.

## Introduction

Cutaneous ureterostomy is a type of urinary diversion involving simple surgical procedures and requiring the least invasive surgical approach, making it suitable for elderly patients and those with a poor performance status. However, cutaneous ureterostomy is generally retroperitoneally constructed. Therefore, the ureter cannot be lifted to the abdominal wall in cases with a limited available ureter length. Thus, selection of a different type of urinary diversion is crucial. We succeeded in constructing a cutaneous ureterostomy in patients with a shorter ureter length by transperitoneal construction using the transverse mesocolon, as was previously described (1).

In the present study, in order to demonstrate that the treatment outcome of transperitoneally constructed cutaneous ureterostomy using the transverse mesocolon is not inferior to that of conventional retroperitoneal cutaneous ureterostomy, a comparative investigation was conducted between patients who were treated with the two procedures during the same time period.

## Materials and methods

### Cases

A total of 160 cases of urinary diversion were performed between March, 2004 and November, 2011 at our institution. Among these, cutaneous ureterostomy was performed in 26 cases and conventional retroperitoneal construction in 15 cases (15 ureters), while transperitoneal construction was performed in 11 cases and 18 ureters. Seven of the transperitoneal ureterocutaneostomy cases were bilateral and all the cases of retroperitoneal ureterocutaneostomy were unilateral. In this study we aimed to describe the transperitoneal ureterocutaneostomy technique using the transverse mesocolon and the outcomes of this procedure in comparison to the conventional technique.

### Technique

**Figure 1.**
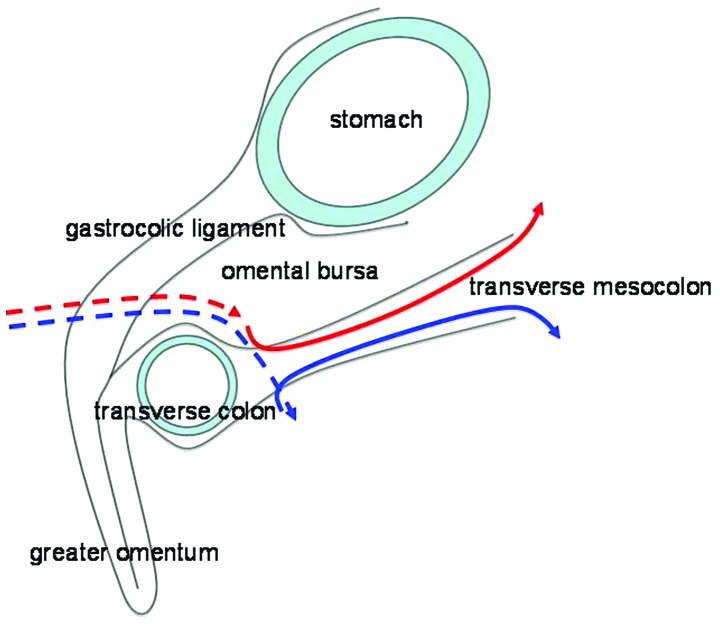
Red arrow, route of the right ureter. Blue arrow, route of the left ureter.

During the procedure, on the right side the transverse colon is caudally mobilized in the same manner as when performing a right radical nephrectomy and the retroperitoneum is incised on the outer side of the descending part of the duodenum. The right ureter is then identified and mobilized. An incision is made into the serosa of the transverse mesocolon close to the area of attachment of the transverse colon, while the great omentum is moved caudally. The subserous part of the transverse mesocolon is detached and a space is created which allows the ureter to pass from this area towards the area of the retroperitoneal incision, similar to creating a tunnel (Fig. 1, red solid line). The right ureter is passed from the root of the transverse mesocolon towards the area to which the transverse colon is attached in this space and the transverse mesocolon is then fixed to the connective tissue surrounding the ureter. Subsequently, the gastrocolic ligament is penetrated from the ventral side towards the area to which the transverse colon is attached, in order to construct a space for the ureter to pass through (Fig. 1, red broken line). The ureter is passed through this area and fixed to its surrounding tissues.

On the left side, the mesocolon is incised between the abdominal aorta and the inferior mesenteric vein at the level of the Treitz suspensory ligament, as during a left radical nephrectomy. The retroperitoneum is reached and the left ureter is identified, mobilized and pulled inside the abdominal cavity. An incision is made on the serosa of the transverse mesocolon near the area to which the transverse colon is attached, while the greater omentum is moved cranially. From this area, the subserous part of the transverse mesocolon is detached towards the mesocolon incision area, similar to creating a tunnel and a space is created for the ureter to pass through (Fig. 1, blue solid line). Subsequently, the gastrocolic ligament and the transverse mesocolon are penetrated from the ventral side towards the area of the small incision near the attachment site of the transverse mesocolon, in order to construct a space for the ureter to pass through (Fig. 1, blue broken line). The ureter is passed from the root of the transverse mesocolon towards the gastrocolic ligament in this space and fixed to its surrounding connective tissue. Subsequently, the ureters are lifted bilaterally to the abdominal wall and a ureteral stoma is constructed. If both the available right and left ureters are short, a ureteral stoma is constructed by a median xiphoid process. Furthermore, for cases in which there is a limited available length for either the right or left ureter, a ureteral stoma may be constructed near the lateral border of the rectus abdominis muscle under the costal arch. An excess 3 cm of the ureter, plus the length from the renal hilus to the abdominal wall, as measured by computed tomography, is a sufficient ureter length for the new construct.

### Statistical analysis

The treatment outcome of cutaneous ureterostomy cases constructed transperitoneally using the transverse mesocolon and that of cases constructed retroperitoneally using the conventional method performed within the same period, were retrospectively evaluated. Decisions regarding adverse events were made based on the Common Terminology Criteria for Adverse Events (CTCAE) v4.0 (2). The Student’s t-test, Chi-square test or Friedman’s test were used for the statistical analysis.

## Results

### Clinical characteristics according to the cutaneous ureterostomy type

The transperitoneal ureterocutaneostomy cases included 5 males and 6 females, with a median age of 81 years (range, 62–94 years) and a median body mass index (BMI) of 22.3 (range, 17.1–26.3). The underlying pathology was bladder cancer in 6 patients, bladder cancer with unilateral ureteral cancer in 2 patients, prostate cancer in 2 patients and vaginal cancer in 1 patient (Table I). Out of the 8 patients with bladder cancer, 1 had stage 0is disease, 1 had stage I disease, 2 had stage II disease, 2 had stage III disease and 2 had stage IV disease, based on the clinical staging system. All 3 patients with prostate or vaginal cancer had stage IV disease. The cutaneous ureterostomy was performed prior to total cystectomy with or without urethrectomy in 7 patients and a cutaneous ureterostomy was performed without cystectomy in 4 cases, 1 of which underwent a colostomy at the same time. Together with total cystectomy, unilateral nephroureterectomy was performed in 2 patients with ureteral cancer. Furthermore, 2 patients had previously undergone unilateral nephroureterectomy or nephrectomy due to ureteral cancer or nephrophthisis. A significant difference was observed regarding age between the groups that underwent the different surgical procedures (P=0.032), with no significant differences regarding the BMI or the follow-up period (P=0.516 and 0.197, respectively).

### Postoperative complications according to the cutaneous ureterostomy type

Ileus developed in 1 (9.1%) bilateral transperitoneal ureterocutaneostomy case and in 1 (6.7%) retroperitoneal cutaneous ureterostomy case, which improved with conservative therapy alone (CTCAE v.4.0 grade 2). No significant difference was observed between the surgical procedures regarding ileus (P=0.827) (Table II).

### Acute pyelonephritis

Acute pyelonephritis developed in 3 (27.3%) transperitoneal ureterocutaneostomy cases, 2 of which were bilateral. The catheter remained in place in all 3 cases. Acute pyelonephritis developed in 8 (53.3%) retroperitoneal ureterocutaneostomy cases, with the catheter remaining in place in all the cases. All the patients showed improvement with the administration of antibiotics alone (CTCAE v.4.0 grade 3). No significant difference was observed between the two surgical procedures regarding the incidence of acute pyelonephritis (P=0.199).

### Catheter-free status according to the cutaneous ureterostomy type

Transperitoneal ureterocutaneostomy was performed in 7 bilateral and 4 unilateral cutaneous ureterostomy cases, totaling 11 cases and 18 ureters. A double-barrel-type stoma was constructed in the center of the upper abdomen in 3 of these cases. All 15 cases undergoing retroperitoneal ureterocutaneostomy were unilateral. No significant difference was observed between surgical procedures regarding the catheter-free status of each individual case (P=0.393) (Table III). No significant difference was observed between surgical procedures regarding the catheter-free status according to the renal units (P=0.525). In addition, no significant difference was observed between surgical procedures regarding the catheter-free status according to the renal units and in those patients in whom it was attempted to obtain a catheter-free status (P=0.437) (Table III).

### Renal function [blood urea nitrogen (BUN) and serum creatinine (Cr) values] according to the cutaneous ureterostomy type (Table IV)

In the transperitoneal ureterocutaneostomy cases, the median rate of change between the serum Cr value prior to surgery and that on the last follow-up day was 97.1% (58.1–148.5) and the median rate of change between the serum Cr value at the early postoperative examination and that on the last follow-up day was 97.1% (78.0–116.3). Moreover, the median rate of change between the BUN value prior to surgery and that on the last follow-up day was 86.4% (58.2–161.0) and the median rate of change between the BUN value at the early postoperative period and that on the last follow-up day was 111.1% (73.4–169.4). No significant difference was observed in the serum Cr or BUN values prior to surgery, at the early postoperative examination or on the last follow-up day.

## Discussion

Cutaneous ureterostomy is usually constructed retroperitoneally in cases where there is a sufficient ureter length available. However, urinary diversion in cases of limited available ureter length and when the use of the digestive tract is preferably avoided has been challenging. To address this problem, we developed a method of construction using a shorter ureter compared to the conventional retroperitoneal ureterocutaneostomy, by transperitoneally constructing the cutaneous ureterostomy using the transverse mesocolon (1). This method is also suitable for cases with anatomical abnormalities, such as extreme obesity, although these cases have a sufficient available ureter length. In this study, in order to prove that the clinical outcome of the transperitoneal ureterocutaneostomy is not inferior to that of the conventional retroperitoneal ureterocutaneostomy, a comparison was conducted between the clinical outcomes of patients who underwent the two different procedures during the same period.

First, postoperative adverse events were investigated. The major advantage of retroperitoneal ureterocutaneostomy is that all operations may be completed in the retroperitoneum. Accordingly, surgical complications accompanying opening the abdominal cavity, such as ileus, may be avoided. In cases in which the transperitoneal technique was applied, the transverse mesocolon was used in addition to opening the abdominal cavity. Therefore, the incidence of ileus was likely to be higher compared to the cases where the transverse mesocolon was not used. However, in the present study, ileus only developed in 1 patient, who recovered with conservative treatment alone and there was no significant difference compared to the retroperitoneal cases (P=0.827). As regards acute pyelonephritis, Yoshimura *et al* reported that the incidence within 3 months following surgery and thereafter was 19.7 and 11.5%, respectively (3), whereas according to Kim *et al*, the incidence was 22.2 and 11.1%, respectively (4). In this study, the incidence of acute pyelonephritis among cases treated with the transperitoneal technique was 27.3% (3 out of the 11 cases) within 1 month from surgery and 18.2% (2 out of the 11 cases) from 3 months onwards. No significant difference was observed between the surgical procedures regarding the occurrence of acute pyelonephritis during the same period (P=0.199); however, the incidence was slightly higher compared to that with conventional techniques (3,4). In all cases, a catheter was present at the onset of acute pyelonephritis in the two surgical procedures. Thus, it is hypothesized that the incidence of acute pyelonephritis may be reduced if a catheter-free status is achieved.

The catheter-free status was then investigated. The rate of catheter-free status was 22.2% (4 out of the 18 ureters) in patients treated with the transperitoneal technique and 13.3% (2 out of the 15 ureters) in those treated with the retroperitoneal technique, demonstrating no significant difference between the surgical procedures (P=0.393). Regarding ureterocutaneostomy, if a single stoma and catheter-free status are achieved bilaterally, this technique may be comparable to an ileal conduit. This is a technique that has received little attention internationally; however, a catheter-free status was achieved in 80–90% of cases by urologists in Japan (5–7). According to a previous study by Terai *et al* regarding double barrel- and barrel-type stoma, 84% of stomas and ipsilateral kidneys and 81% of the contralateral kidneys achieved a catheter-free status; therefore, a catheter-free status was achieved in 79% of all cases (6). However, for cases exhibiting stoma stenosis, rendering ureteral catheter placement essential, the quality of life of the patient may be compromised due to the possible onset of complications, such as urinary tract infections and renal dysfunction. In the present study, there was no significant difference between the surgical procedures regarding the median follow-up period (P=0.197). However, the median follow-up period of transperitoneal ureterocutaneostomy cases was only 8.3 months, almost half of that observed in retroperitoneal ureterocutaneostomy cases, which was 20.1 months. The purpose of the transperitoneal technique in several cases was to secure the urinary tract and ease the symptoms of the patient during palliative care. Accordingly, this procedure was a type of urinary diversion performed during the palliative operation; therefore, a catheter-free status was not our intent in all cases. Furthermore, securing a sufficient amount of urine is necessary at the early postoperative period for cases where securing the urinary tract is required for chemotherapy; therefore, a catheter-free status was not attempted in these cases. Moreover, certain chemotherapeutic regimens require placement of a catheter and attempting to achieve a catheter free-status may lead to a delay in the initiation of chemotherapy, a decrease in renal function and the administration of an insufficient volume of anticancer drugs, due to the hydronephrosis that occurs at least temporarily. The risk of pyelonephritis may also increase and renal function may decrease in the long-term. Therefore, we did not attempt to achieve a catheter-free status in all the cases, since our priority was to ensure sufficient renal function and allow the administration of adequate volume of anticancer drugs during the early postoperative period. We only attempted to provide a catheter-free status in cases with predicted long-term survival.

Due to the the reasons mentioned previously, the catheter-free status was attempted in 6 (8 ureters, since 2 cases were bilateral) out of the 11 cases (18 ureters) treated by transperitoneal cutaneous ureterostomy. Among these, a catheter-free status was achieved in 4 cases and 4 ureters (1 case was a bilateral cutaneous ureterostomy with a catheter-free status achieved on one side). Accordingly, any evaluation considering only the cases in which a catheter-free status was attempted revealed a success rate of 50% (4 out of 8 ureters).

A catheter-free status was not attempted in all cases of retroperitoneal cutaneous ureterostomy due to the same reasons mentioned above. Out of the 15 cases (15 ureters) who underwent retroperitoneal ureterocutaneostomy, a catheter-free status was attempted in 7 cases and 7 ureters. Of these, a catheter-free status was achieved in 2 cases and 2 ureters (28.6%). These results lead to the conclusion that a catheter-free status is feasible even when the cutaneous ureterostomy is transperitoneally constructed. Furthermore, no significant difference was observed between the surgical procedures regarding the catheter-free status. However, the rate of attaining a catheter-free status was lower among the conventional retroperitoneal ureterocutaneostomy cases. Therefore, it is considered necessary to conduct a ureterostomy according to the Toyoda method (5), which was also adopted by Terai *et al* (6), or the Hirokawa method (7), in order to avoid the need for an indwelling catheter due to future stoma stenosis.

As regards renal function, no significant changes (BUN and serum Cr values) were observed in the transperitoneal ureterocutaneostomy cases at the postoperative, early postoperative (1 month after surgery) and the final follow-up examinations (P=0.739 and 0.078). Therefore, there was no significant effect on the renal function when the ureter was run transperitoneally using the transverse colon. The incidence of acute pyelonephritis was slightly higher with the transperitoneal technique, compared to studies on conventional retroperitoneal ureterocutaneostomy, with a lower rate of catheter-free status. However, renal function did not decrease between the preoperative and postoperative examinations. Adopting a procedure to avoid the stenosis of the stoma may likely lead to an increase in the catheter-free status rate and decrease the occurrence of acute pyelonephritis.

In conclusion, transperitoneal cutaneous ureterostomy using the transverse mesocolon is a surgical procedure that should be considered for urinary diversions in cases with a limited available ureter length and in which use of the digestive tract is preferably avoided.

## Figures and Tables

**Table I.** Clinical characteristics of the patients according to the cutaneous ureterostomy type.

Characteristics	Transperitoneal	Retroperitoneal	P-value
Total no. of patients	11	15	
Gender			
Male	5	10	
Female	6	5	
Age (years)			
Median (range)	81 (62–94)	71 (51–84)	0.032
80>	4	11	
80≤	7	4	
BMI			
Median (range)	22.3 (17.1–26.3)	22.8 (18.4–26.5)	0.516
Underlying disease			
Bladder cancer	6	5	
Bladder cancer + ureteral cancer	2	2	
Bladder cancer + renal pelvic cancer	0	1	
Bladder cancer + prostate cancer	0	2	
Bladder cancer + atrophic kidney	0	3	
Prostate cancer	2	0	
Prostate cancer + atrophic kindey	0	2	
Vaginal cancer	1	0	
Operation			
Ureterostomy alone	3	1	
+ cystectomy	5	5	
+ cystectomy + nephroureterectomy	2	8	
+ colostomy	1	0	
+ pelvic exenteration + nephroureterectomy + colostomy	0	1	
Follow-up period (months)			
Median (range)	8.3 (1.0–58.7)	20.1 (3.5–83.3)	0.197
Survival			
Alive	4	6	
Deceased	7	9	

BMI, body mass index.

**Table II.** Postoperative complications according to cutaneous ureterostomy type.

Complications	Transperitoneal	Retroperitoneal	P-value
Ileus	1	1	0.827
Acute	3	8	0.199
pyelonephritis			
within 3 months	3	8	
after 3 months	2	7	

**Table III.** Catheter-free status according to the cutaneous ureterostomy type.

Type	Transperitoneal	Retroperitoneal	P-value
Ureter			
Total	18	15	
Right	2	8	
Left	2	7	
Bilateral	7	0	
Stomal side			
Right	5	8	
Left	3	7	
Middle	3	0	
Catheter-free (cases)			
Yes	3	2	0.393
No	8	13	
Catheter-free (renal units)			
Yes	4	2	0.525
No	14	13	
Attempted catheter-free (renal units)			
Yes	4	2	0.437
No	4	5	

**Table IV.** Renal function according to the cutaneous ureterostomy type.

Renal functions	Transperitoneal	Retroperitoneal
Serum creatinine (mg/dl), median (range)		
Prior to operation	0.9 (0.6–1.5)	1.1 (0.6–1.9)
One month after operation	0.8 (0.6–1.5)	1.2 (0.6–1.6)
Last follow-up	0.9 (0.5–1.5)	1.2 (0.5–2.8)
P-value	0.739	0.104
Blood urea nitrogen (mg/dl), median (range)		
Prior to operation	23.7 (12.3–35.3)	17.5 (11.0–40.7)
One month after operation	19.3 (10.0–26.1)	15.4 (10.7–36.5)
Last follow-up	19.2 (13.2–32.7)	20.1 (8.2–44.6)
P-value	0.078	0.022

## References

[1] Furubayashi N, Nakamura M, Hishikawa K, Fukuda A, Matumoto T, Hasegawa Y (2012). Cutaneous ureterostomy using the transverse mesocolon. Int J Urol.

[2] Common Terminology Criteria for Adverse Events (CTCAE) http://ctep.cancer.gov/protocolDevelopment/electronic_applications/ctc.htm#ctc_40.

[3] Yoshimura K, Maekawa S, Ichioka K, Terada N, Matsuta Y, Okubo K, Arai Y (2001). Tubeless cutaneous ureterostomy: the Toyoda method revisited. J Urol.

[4] Kim CJ, Wakabayashi Y, Sakano Y, Johnin K, Yoshiki T, Okada Y (2005). Simple technique for improving tubeless cutaneous ureterostomy. Urology.

[5] Toyoda Y (1977). A new technique for catheterless cutaneous ureterostomy. J Urol.

[6] Terai A, Yoshimura K, Ueda N, Utsunomiya N, Kohei N, Arai Y (2006). Clinical outcome of tubeless cutaneous ureterostomy by the Toyoda method. Int J Urol.

[7] Hirokawa M, Iwasaki A, Yamazaki A, Asakura S, Nozaki A, Yamagishi T (1989). Improved technique of tubeless cutaneous ureterostomy and results of permanent urinary diversion. Eur Urol.

